# Investigating host dependence of xylose utilization in recombinant *Saccharomyces cerevisiae* strains using RNA-seq analysis

**DOI:** 10.1186/1754-6834-6-96

**Published:** 2013-07-06

**Authors:** Xueyang Feng, Huimin Zhao

**Affiliations:** 1 Department of Chemical and Biomolecular Engineering, Institute for Genomic Biology, Urbana, USA; 2 Departments of Chemistry, Biochemistry, and Bioengineering, University of Illinois at Urbana-Champaign, Urbana IL 61801, USA

**Keywords:** RNA-seq, Xylose metabolism, Transcriptional factors, *S. cerevisiae*, Pathway switch, Host-specific response

## Abstract

**Background:**

Xylose-based ethanol production by recombinant *S. cerevisiae* is of great interest to basic and applied bioenergy research. By expressing three different fungal pathways in two *S. cerevisiae* hosts respectively, we found that the xylose utilization efficiency by recombinant *S. cerevisiae* depends not only on the choice of xylose pathway but also on the choice of host, exhibiting an obvious host or context dependence. To investigate molecular mechanisms of this context dependence, we applied RNA-seq analysis in this study for a systematic characterization of the xylose utilization via different pathways in different *S. cerevisiae* hosts.

**Results:**

Based on the RNA-seq analysis, the transcripts that were regulated during xylose utilization have been identified. Three transcription factors involved in regulation of amino acid metabolism, responses to oxidative stresses, and degradation of aggregated proteins, respectively, were found to participate in xylose metabolism regulation regardless of which pathway was expressed and which host the xylose pathway was expressed in. Nine transcription factors, involved in homeostasis, regulation of amino acid metabolism, and stress responses, were identified as the key modules responsible for the host-specific responses to the same xylose pathway. In addition, the transcriptional regulations of xylose utilization in different yeast hosts were compared to two reference regulation patterns, which indicated that diverse regulation strategies were adopted by different hosts for improved xylose utilization.

**Conclusions:**

This study provides the first transcriptomic study of the host dependence of xylose utilization in *S. cerevisiae*. Both the conserved regulatory modules for xylose metabolism and the key modules responsible for host dependence were identified. As indicated by the functions of the conserved transcription factors involved in xylose metabolism regulation, the xylose utilization in recombinant *S. cerevisiae* may be affected by both carbohydrate metabolism regulation and stress responses. Based on the comparison of transcriptional regulation patterns, the metabolic optimizations of xylose utilization in different hosts went toward different directions, which may explain the host dependence observed in this study. The knowledge revealed by this study could provide valuable insights towards the improvement of metabolic engineering strategies for cellulosic ethanol production.

## Background

Engineering *S. cerevisiae* to utilize xylose for ethanol production is of great interest to the biofuel industry because it can reduce the cost of feedstock for bioethanol production and substantially minimize the emission of greenhouse gases [1,2]. To achieve this objective, a heterologous xylose pathway, consisting of xylose reductase (XR), xylitol dehydrogenase (XDH), and xylulose kinase (XKS), is usually functionally expressed in *S. cerevisiae*[3-5], followed by the optimization of xylose fermentation behaviors via a series of metabolic engineering approaches such as promoter engineering [6,7] and evolutionary engineering [8].

Previously, we engineered two *S. cerevisiae* hosts, namely CTY and INVSc1, to efficiently utilize xylose for bioethanol production by using the COMPACTER approach [6]. In brief, the promoter strengths of XR, XDH, and XKS have been tuned in each host respectively to generate a library of mutated xylose pathways followed by high-throughput screening. Two optimized pathways, one from the CTY host (CTYp) and the other from the INVSc1 host (INVp), were found to have superior performance compared to the wild-type pathway (WT) (i.e., without optimization of promoter strengths) (Table 1). Interestingly, switching the optimized pathway from the original host into the other host led to poorer fermentation profiles. For example, the xylose pathway optimized in the CTY host (CTYp) cannot achieve the equally high ethanol yield or xylose uptake rate in the INVSc1 host (i.e., INV-CTYp) as that in the CTY host (i.e., CTY-CTYp). The similar mismatch was also found for the xylose pathway optimized in the INVSc1 host (INVp), which led to lower ethanol yield and xylose uptake rate when expressed in the CTY host (CTY-INVp) than in the INVSc1 host (INV-INVp). Therefore, the xylose metabolism of recombinant *S. cerevisiae* depends not only on the pathway but also the host.

**Table 1:** Physiological analysis of CTY and INV with different xylose pathways

	**CTY-WT**	**CTY-CTYp**	**CTY-INVp**	**INV-WT**	**INV-CTYp**	**INV-INVp**
μ (h^-1^)	0.007 ± 0.005	0.024 ± 0.002	0.016 ± 0.006	0.015 ± 0.003	0.028 ± 0.002	0.031 ± 0.006
*q*_*xylose*_ (mmol/g/h)	0.16 ± 0.02	0.60 ± 0.02	0.44 ± 0.00	0.28 ± 0.01	0.43 ± 0.05	0.55 ± 0.04
*Y*_*Xylitol*_ (g/g)	0.04 ± 0.00	0.12 ± 0.01	0.00 ± 0.00	0.05 ± 0.00	0.07 ± 0.00	0.02 ± 0.01
*Y*_*Glycerol*_ (g/g)	0.00 ± 0.00	0.00 ± 0.00	0.05 ± 0.04	0.06 ± 0.00	0.06 ± 0.00	0.05 ± 0.00
*Y*_*Acetate*_ (g/g)	0.00 ± 0.00	0.00 ± 0.00	0.00 ± 0.00	0.00 ± 0.00	0.00 ± 0.00	0.02 ± 0.00
*Y*_*EtOH*_ (g/g)	0.00 ± 0.00	0.25 ± 0.01	0.00 ± 0.00	0.15 ± 0.06	0.21 ± 0.01	0.29 ± 0.04

**Figure 1:**
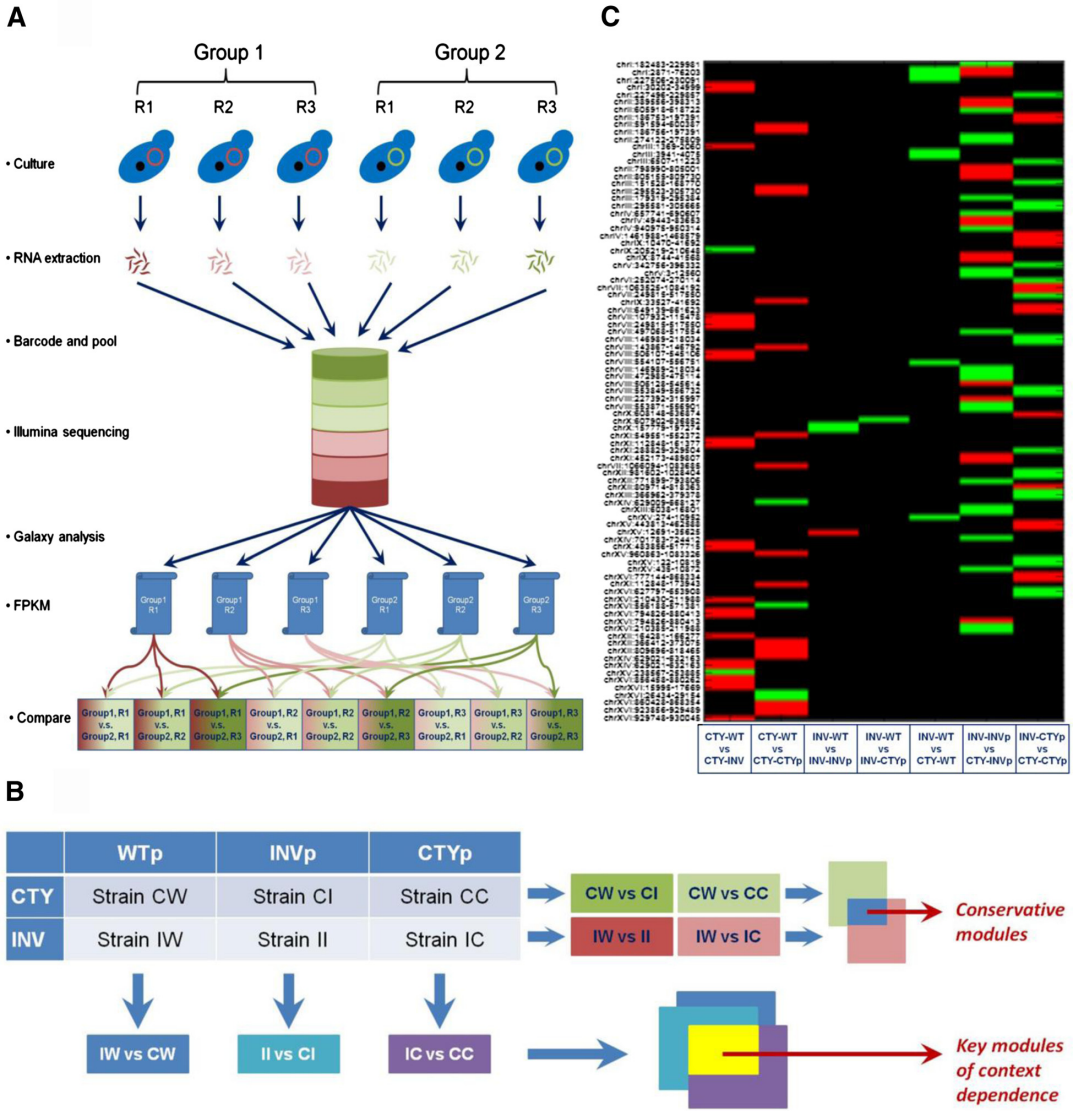
**Overview of transcriptomics analysis of xylose metabolism in *S. cerevisiae*.** (**A**) Flowchart of RNA-seq analysis; (**B**) The roadmap for finding the conserved regulatory modules in xylose metabolism and the key regulatory modules in host dependence, and (**C**) the summary of transcriptional behaviors of the CTY and INV hosts in response to different xylose pathways. In each comparison of gene expressions, the control group (e.g., CW in CW vs. CI) was compared to the experimental group (e.g., CI in CW vs. CI). The black, red and green colors in (**C**) indicate no significant change of gene expressions, decreased gene expressions in the experimental group, and increased gene expressions in the experimental group, respectively.

Towards an in-depth and mechanistic understanding of such host dependence, we used RNA-seq analysis to investigate and compare the transcriptional responses of a series of recombinant *S. cerevisiae* strains to xylose metabolism. Basically, three different xylose pathways (i.e., WT, CTYp and INVp) were functionally expressed in two hosts of *S. cerevisiae* (i.e., CTY and INVSc1), which generated six recombinant strains in total. Specifically, we aimed to find the answers to two questions about the xylose metabolism in *S. cerevisiae*: 1) what are the conserved modules that are involved in xylose metabolism regulations; and 2) what are the key modules that lead to the host dependence. By systematically grouping the differentially expressed genes by the transcription factors (TFs) and comparing the profiles of TFs in the CTY and INVSc1 hosts respectively (Figure 1), we found three TFs were used by both hosts for regulating xylose metabolism. Similarly, nine TFs were identified as potential key modules that may participate in the host dependence of xylose metabolism. To the best of our knowledge, this is the first study that systematically evaluates the transcriptional behaviors of host dependence in sugar metabolism of yeasts.

## Results

### Physiology of host dependence in xylose metabolism of *S. cerevisiae*

To optimize xylose utilization, the fungal xylose pathway has been independently engineered in two *S. cerevisiae* hosts, CTY and INVSc1, by tuning the promoter strengths of three key genes (i.e., XR, XDH, and XKS). Two optimized pathways were selected respectively in either CTY (i.e., CTYp) or INVSc1 (i.e., INVp) based on their improved performance compared to the wild-type pathway (WT). In general, in the CTY host, the strain with the optimized pathway (i.e., CTY-CTYp) enhanced the xylose uptake rate by nearly three fold (from 0.16 to 0.60 mmol/g DCW/h, Table 1) compared to the parent strain with the wild-type pathway (i.e., CTY-WT). While no ethanol can be produced by CTY-WT, the xylose-based ethanol yield can reach as high as 0.25 g/g in CTY-CTYp. Similarly, in the INVSc1 host, the xylose uptake rate and ethanol yield were increased by 94% and 93% respectively in the strain with the optimized pathway (i.e., INV-INVp) compared to the parent strain with the wild-type pathway (i.e., INV-WT).

However, upon switching the INVp pathway (i.e., the pathway optimized in the INVSc1 host) from the INVSc1 host to the CTY host, the resultant CTY-INVp strain has a 25% decrease in the xylose uptake rate and nearly 100% decrease in ethanol production compared to those of INV-INVp. Similarly, a 28% decrease in xylose uptake rate and a 16% decrease in ethanol yield were found in another pathway-switched strain, INV-CTYp, compared to those of CTY-CTYp. In general, despite of the similar genomic background between the two *S. cerevisiae* hosts and same engineering approaches applied to optimize the xylose metabolism, the metabolic responses of the CTY and INVSc1 hosts to the same xylose pathway were not the same, which indicates the host or context dependence during the xylose utilization in recombinant *S. cerevisiae* strains.

### Overview of transcriptomics analysis

**Figure 2:**
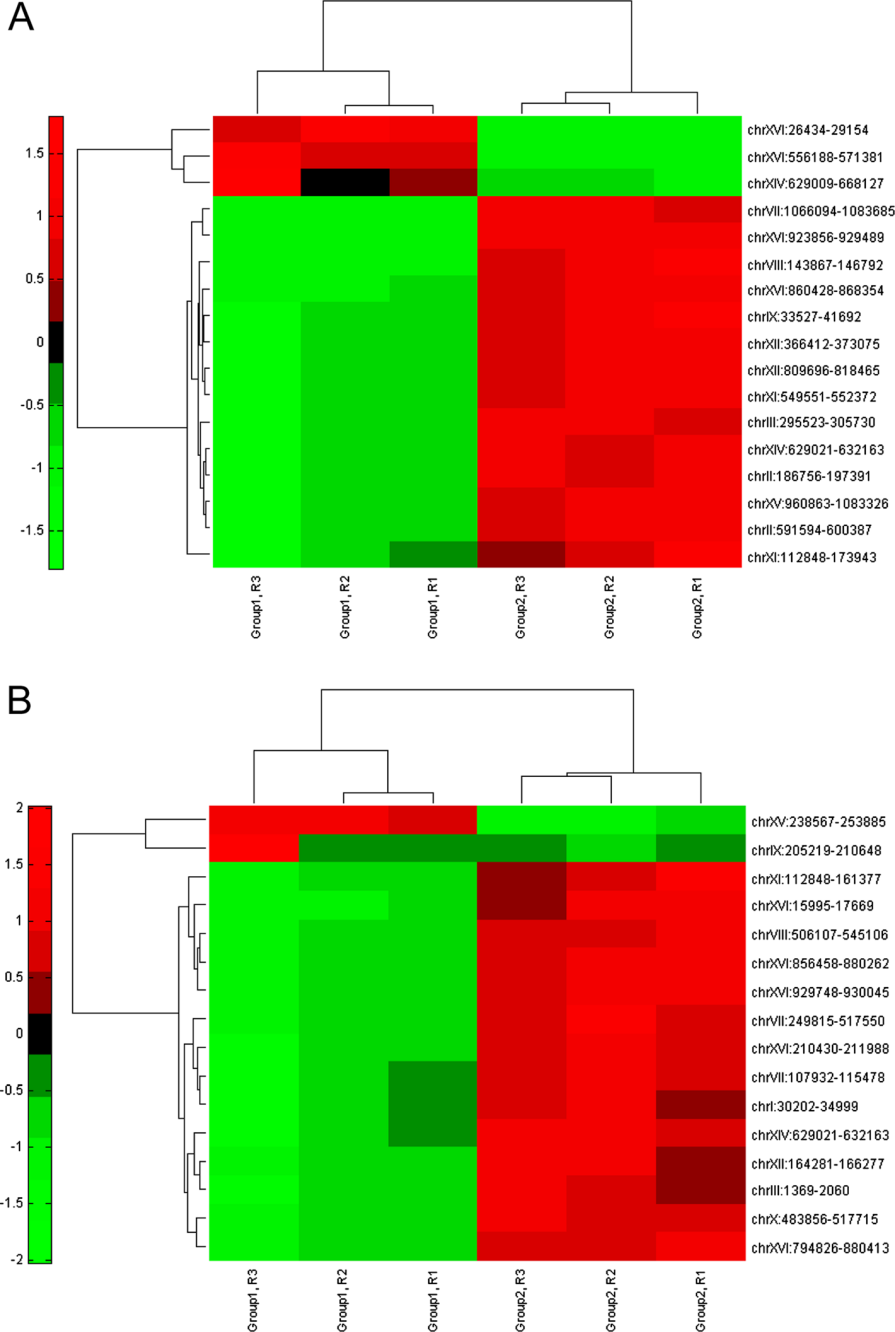
**Cluster analysis of transcriptional responses of CTY host to xylose metabolism.** (**A**) Cluster analysis of transcriptional behaviors between CTY-WT and CTY-CTYp, in which group 1 and group 2 indicate CTY-WT and CTY-CTYp respectively, and R1, R2, and R3 indicate three biological replicates; (**B**) Cluster analysis of transcriptional behaviors between CTY-WT and CTY-INVp, in which group 1 and group 2 indicate CTY-WT and CTY-INVp respectively, and R1, R2, and R3 indicate three biological replicates.

**Figure 3:**
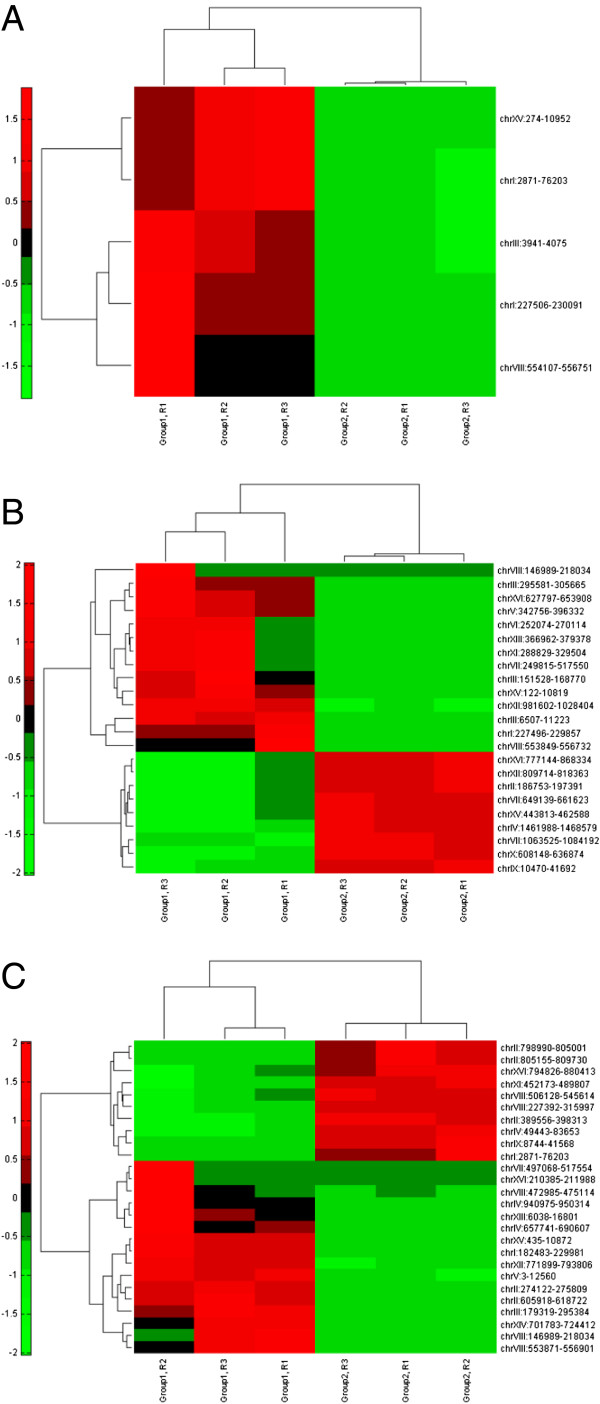
**Cluster analysis of transcriptional responses in host dependence.** (**A**) Cluster analysis of transcriptional behaviors between INV-WT and CTY-WT, in which group 1 and group 2 indicate INV-WT and CTY-WT, respectively, and R1, R2, and R3 indicate three biological replicates; (**B**) Cluster analysis of transcriptional behaviors between INV-CTYp and CTY-CTYp, in which group 1 and group 2 indicate INV-CTYp and CTY-CTYp respectively, and R1, R2, and R3 indicate three biological replicates; (**C**) Cluster analysis of transcriptional behaviors between INV-INVp and CTY-INVp, in which group 1 and group 2 indicate INV-INVp and CTY-INVp, respectively, and R1, R2, and R3 indicate three biological replicates.

To systematically characterize the transcriptional responses of the CTY and INV hosts to different xylose pathways, the RNA-seq analysis with over 442 million sequence reads in total was finished for 18 samples, which includes six recombinant strains (CTY-WT, CTY-CTYp, CTY-INVp, INV-WT, INV-CTYp, and INV-INVp) with three biological replicates respectively. To identify the transcripts that have significantly different expression levels between the control group and the experimental group, the Cuffdiff program was used with the default parameter setting. The advantage of using Cuffdiff’s count-based differential expression analysis is that the error introduced by the isologues of the genes can be well corrected, which provides more accurate and in-depth analysis of the transcriptional behaviors [9]. However, as reported previously [10], the noise among different biological replicates, resulting from sample heterogeneity, genetic polymorphism, and changes in mRNA levels within cells and among individuals due to genotype-environment interactions as well as other factors, could be the greatest source of variations during transcriptional studies. In this study, we have carefully controlled the experimental workflow from batch culture of xylose fermentation to total RNA extractions, with the correlation of the overall transcriptome readouts among biological replicates reaching as high as 0.996. However, no transcript stood out as differentially expressed between the control group and the experimental group when pooling the expression data of more than two biological replicates together for Cuffdiff analysis. To remove the noise arising from the variations among biological replicates, we pursued for qualitative identification of differentially expressed transcripts by designing the flowchart as shown in Figure 1. In general, instead of pooling all the data from the triplicates together in Cuffdiff, we used one replicate from the control group and one replicate from the experimental group as the input for Cuffdiff, and exhausted all the possible comparisons between the control group and the experimental group (i.e., nine comparisons from three replicates from the control group and three replicates from the experimental group). From each of the Cuffdiff comparisons, certain transcripts would be identified as significantly up-/down- regulated. Then, we chose the cut-off values to pick the transcripts that have consistent behaviors among the comparisons. As one will expect, more transcripts would be picked with lower cut-off values (Additional file 1: Figure S1). In this study, we chose the highest cut-off value (i.e., n = 9) to find the transcripts that can be definitely identified to be differentially expressed. The FPKM values from these selected transcripts were then used for transcriptional analysis (Figures 2 and 3) and gene ontology (GO) analysis (Tables 2 and 3). The RNA-seq based fold changes of the three heterologous genes in the xylose pathway (i.e., XR, XDH, and XKS) were compared to the qPCR results previously reported [6], showing positive correlation with a Pearson correlation coefficient of 0.91.

**Table 2:** Gene ontology analysis of transcriptional responses of CTY and INVSc1 hosts to xylose metabolism

**GO ID**	**Description**	**CW vs CC**	**CW vs CI**	**IW vs IC**	**IW vs II**
**Up-regulated**					
**GO:0000902**	cell morphogenesis	FKS1			
**GO:0005975**	carbohydrate metabolic process	ERR1, FKS1, GPH1, GPM1, IMA1, MAL12, PYK2, SUC2, CIT1	ALG13, ALG2, CIT1, CWH41, GPH1, KRE6, PYC1, STT3		
**GO:0006091**	generation of precursor metabolites and energy	ATF1, ERR1, FRE3, GPH1, GPM1, PYK2, CIT1	CIT1, GPH1, OLE1		
**GO:0006457**	protein folding		CPR7		
**GO:0006486**	protein glycosylation		ALG2, CWH41, STT3		
**GO:0006520**	cellular amino acid metabolic process	GDH1, CIT1	ASN1, CIT1, GDH3, LEU1, MET13, MET16, TRP5, YGR012W		
**GO:0006629**	lipid metabolic process	MCR1, YPC1	ALG13, ALG2, ECT1, ERG26, ERG4, MET13, MET16, OLE1, POX1		
**GO:0006811**	ion transport	FRE3			
**GO:0006873**	cellular ion homeostasis	FRE3			
**GO:0006897**	endocytosis	FKS1			
**GO:0006979**	response to oxidative stress	AHP1, MCR1			
**GO:0008033**	tRNA processing		PUS2		
**GO:0008380**	RNA splicing	MNE1			
**GO:0009311**	oligosaccharide metabolic process	IMA1, MAL12, SUC2	ALG2, CWH41		
**GO:0009451**	RNA modification		PUS2		
**GO:0018193**	peptidyl-amino acid modification		CPR7		
**GO:0023052**	signaling	PDE2			
**GO:0042221**	response to chemical stimulus	AHP1, MCR1	MNL1		
**GO:0045333**	cellular respiration	CIT1	CIT1		
**GO:0051049**	regulation of transport	FKS1			
**GO:0051603**	proteolysis involved in cellular protein catabolic process		ATE1, MNL1		
**GO:0051604**	protein maturation		KEX1		
**GO:0051186**	cofactor metabolic process	ACH1, ALD4, FDH1, CIT1	CIT1, HEM2, MET13, NMA2, NPY1, PNC1, PYC1		
**GO:0055086**	nucleobase-containing small molecule metabolic process	ALD4, FDH1	NMA2, NPY1, PNC1, PYC1		
**GO:0071554**	cell wall organization or biogenesis		CWH41, KRE6		
Un-identified, up-regulated genes*		POT1, SDH1	BAT1		
**Down-regulated**					
**GO:0005975**	carbohydrate metabolic process	CIT3			***RPE1***
**GO:0006091**	generation of precursor metabolites and energy	CIT3			
**GO:0006470**	protein dephosphorylation			***YMR1***	
**GO:0006520**	cellular amino acid metabolic process		***GSH2***	***CPA2, URA8***	***URA2***
**GO:0006605**	protein targeting	ACC1			
**GO:0006629**	lipid metabolic process	ACC1, ARE2, CIT3, LRO1		***URA8, YJR107W, YMR1***	***LCB3***
**GO:0006873**	cellular ion homeostasis			***SOD1***	
**GO:0006979**	response to oxidative stress			***SOD1***	
**GO:0006997**	nucleus organization	ACC1			
**GO:0023052**	signaling				***LCB3***
**GO:0042221**	response to chemical stimulus			***SOD1***	
**GO:0045333**	cellular respiration	CIT3			
**GO:0051169**	nuclear transport	ACC1			
**GO:0051186**	cofactor metabolic process	ACC1, CIT3			***RPE1***
**GO:0055086**	nucleobase-containing small molecule metabolic process	ACC1, URK1		***ADO1, URA8***	***RPE1, URA2***
**GO:0071554**	cell wall organization or biogenesis			***SOD1***	
Un-identified, down-regulated genes*					***NIT2***
**Summary**	**Up-regulated**	**22**	**30**	**0**	**0**
	**Down-regulated**	**5**	**1**	**6**	**4**

Note: the genes marked as bold and italic have over 10 fold changes of expression, while the others have less than 10 fold changes of expression.

*: some of the genes that were down-/up- regulated cannot be mapped into GO slim files.

**Table 3:** Gene ontology analysis of transcriptional responses in host dependence

**GO ID**	**Description**	**IW vs CW**	**IC vs CC**	**II vs CI**
**Up-regulated**				
GO:0000902	cell morphogenesis		FKS1	
GO:0005975	carbohydrate metabolic process		ALG8, FKS1, GPH1, ***IMA1***, ***IMA3***, KRE6, ***MAL12***, ***SUC2***	***CDC19***, GPH1, IMA3, KRE6, ***MAL32***, ***SUC2***
GO:0006091	generation of precursor metabolites and energy		CYT1, GPH1	***ACS1***, ***CDC19***, GPH1
GO:0006325	chromatin organization			ACS1
GO:0006470	protein dephosphorylation		***YMR1***	
GO:0006486	protein glycosylation		ALG8	
GO:0006520	cellular amino acid metabolic process		ASN1, ***CPA2***, PDC6, ***URA8***, ***YIL168W***	ASN1, DYS1, ***GCV3***, GDH2, ***GDH3***, MET16, ***YIL168W***
GO:0006629	lipid metabolic process		ALG8, ***URA8***, ***YJR107W***, ***YMR1***	ERG7, FOX2, MET16
GO:0006766	vitamin metabolic process		***BIO2***	
GO:0006873	cellular ion homeostasis		***SOD1***	
GO:0006897	endocytosis		FKS1	
GO:0006979	response to oxidative stress		CTT1, ***SOD1***	
GO:0007005	mitochondrion organization			PPE1
GO:0009311	oligosaccharide metabolic process		ALG8, ***IMA1***, ***IMA3***, ***MAL12***, ***SUC2***	***IMA3***, ***MAL32***, ***SUC2***
GO:0016570	histone modification			***ACS1***
GO:0018193	peptidyl-amino acid modification			***ACS1***, DYS1
GO:0032543	mitochondrial translation			PPE1
GO:0042221	response to chemical stimulus		CTT1, ***SOD1***	MNL1
GO:0043543	protein acylation			***ACS1***
GO:0045333	cellular respiration		CYT1	
GO:0051049	regulation of transport		FKS1	
GO:0051186	cofactor metabolic process		ACH1, ***BIO2***	***ACS1***, QNS1
GO:0051603	proteolysis involved in cellular protein catabolic process			MNL1
GO:0055086	nucleobase-containing small molecule metabolic process		***ADO1***, ***URA8***	QNS1
GO:0070271	protein complex biogenesis			***CYC3***
GO:0071554	cell wall organization or biogenesis		KRE6, ***SOD1***	KRE6
Un-identified, up-regulated genes*			***NIT1 POT1***	***BDH1*** NIT1 ***POT1***
**Down-regulated**				
GO:0000746	conjugation		CHS5	
GO:0000910	cytokinesis			CHS5
GO:0005975	carbohydrate metabolic process	CDC19	ALG13, ALG2, CWH41, ***DOG1***, ***DOG2***, FBA1, HXK1, ***INM1***, PYC1, STT3	CHS5, ***DOG1***, ***DOG2***, ***GAL7***, ***INM1***, ***PGI1***, ***RBK1***
GO:0006091	generation of precursor metabolites and energy	ACS1, CDC19	***COX6***, FBA1, HXK1, OLE1	***COX6***, ***PGI1***, ***TRX3***
GO:0006281	DNA repair		YNK1	
GO:0006325	chromatin organization	ACS1		
GO:0006457	protein folding			***CPR4***
GO:0006486	protein glycosylation		ALG2, CWH41, STT3	
GO:0006520	cellular amino acid metabolic process	GCV3, GDH3	CAR2, ***GLN1***, IRC7, LEU1, MET13, ***PUT2***, ***THR1***, TRP5, YGR012W	ALT2, LYS9, ***PUT2***, ***THR1***, ***THR4***, YGR012W
GO:0006629	lipid metabolic process		ALG13, ALG2, ECT1, ERG26, ERG4, INM1, IRC7, MET13, ***NCP1***, OLE1, ***THR1***	***ARE1***, ***ATG15***, ECT1, ***INM1***, MVD1, ***NCP1***, ***THR1***, YAT1
GO:0006974	response to DNA damage stimulus		YNK1	
GO:0006979	response to oxidative stress			***TRX3***
GO:0008033	tRNA processing		PUS2	
GO:0008643	carbohydrate transport		HXK1	
GO:0009311	oligosaccharide metabolic process		ALG2, CWH41	
GO:0009451	RNA modification		PUS2	
GO:0010324	membrane invagination			***ATG15***
GO:0016570	histone modification	ACS1		
GO:0018193	peptidyl-amino acid modification	ACS1		***CPR4***
GO:0042221	response to chemical stimulus			***TRX3***
GO:0043543	protein acylation	ACS1		
GO:0043934	sporulation			CHS5
GO:0045333	cellular respiration		***COX6***	***COX6***
GO:0048193	Golgi vesicle transport			CHS5
GO:0051186	cofactor metabolic process	ACS1	HEM2, MET13, NMA2, NPY1, PNC1, PYC1	MVD1, NMA1, NMA2, ***PGI1***
GO:0051603	proteolysis involved in cellular protein catabolic process		ATE1	
GO:0051604	protein maturation		ATG15	
GO:0055086	nucleobase-containing small molecule metabolic process	***IMD2***	***IMD2***, IMD3, NMA2, NPY1, PNC1, ***PRS2***, PYC1, YNK1	***IMD2***, NMA1, NMA2, ***PGI1***
GO:0070271	protein complex biogenesis		CYC3	
GO:0071554	cell wall organization or biogenesis		CWH41, ***PRS2***	CHS5
Un-identified, down-regulated genes*		BDH1 ***AAD15***	***AAD15***	PHO11 ***AAD15 FEN1***
**Summary**	**Down-regulated**	**8**	**36**	**29**
	**Up-regulated**	**0**	**23**	**24**

Note: the genes marked as bold and italic have over 10 fold changes of expression, while the others have less than 10 fold changes of expression.

*: some of the genes that were down-/up- regulated cannot be mapped into GO slim files.

### Transcriptional responses of *S. cerevisiae* hosts to xylose utilization

In the CTY host, 17 and 16 transcripts were identified to have different expression levels in CTY-CTYp and CTY-INVp, respectively, as compared to those from CTY-WT (Figures 1C and 2). A further annotation of genes to the transcripts revealed that 22 genes were up-regulated and 5 genes were down-regulated in CTY-CTYp, while 30 genes were up-regulated and 1 gene was down-regulated in CTY-INVp (Table 2). Based on GO analysis, while the metabolic processes that were transcriptionally regulated were not exactly the same in response to different xylose pathways, several biological processes involved in central carbon and energy metabolisms, such as carbohydrate metabolic process (GO:0005975), nucleobase-containing small molecule metabolic process (GO:0055086), lipid metabolic process (GO:0006629), cofactor metabolic process (GO:0051186), and cellular amino acid metabolic process (GO:0006520), were found to be transcriptionally regulated regardless of which xylose pathway was utilized in the CTY host.

In the INVSc1 host, only 1 transcript in INV-CTYp and 2 transcripts in INV-INVp were identified to be differentially expressed compared to those of INV-WT (Figure 1C and Additional file 1: Figure S2). Based on the genome annotation, all of the 6 genes included in the transcripts of INV-CTYp and the 4 genes included in the transcripts of INV-INVp were found to be down-regulated. The GO analysis indicated that three biological processes, including nucleobase-containing small molecule metabolic process (GO:0055086), lipid metabolic process (GO:0006629), and cellular amino acid metabolic process (GO:0006520), were involved in the transcriptional regulation of xylose metabolism regardless of which xylose pathway was used in the INVSc1 host.

**Figure 4:**
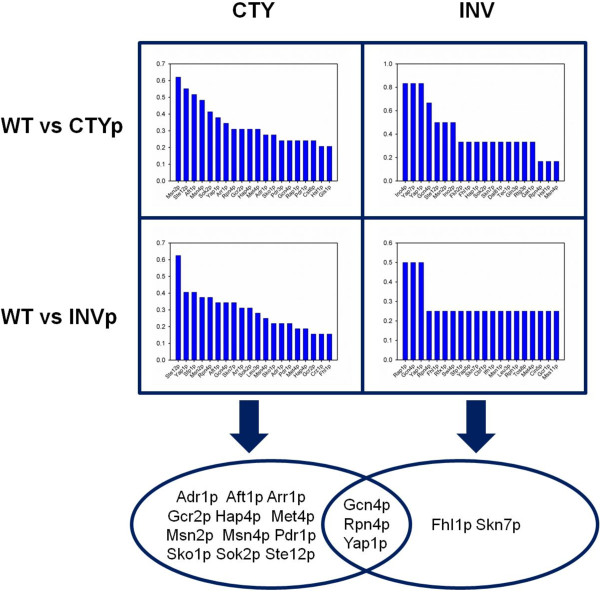
**TF profiles shown as the percentage of genes regulated by the top 20 TFs relative to the total number of genes involved in xylose metabolism regulations.** The highlighted TFs in the oval indicate the ones that appeared in all of the xylose regulation experiments in the hosts of CTY and INV, respectively. Notably, Gcn4p, Rpn4p, and Yap1p were found to be involved in all of the xylose metabolism regulations regardless of the choice of the host.

The exhibition of complex genetic responses of *S. cerevisiae* to the environment was largely due to the transcription factors (TF) that govern the way of controlling the flow of genetic information from DNA to mRNA [11]. To uncover the regulation machinery of xylose metabolism, we next performed TF analysis by searching for the TFs that were reported to most likely regulate the genes involved in xylose utilization (Figure 4). To avoid the biased researching for the general TFs that can potentially regulate nearly all of the genes in *S. cerevisiae*, in this study, we only considered the TF-gene regulations supported by published data. For each TF analysis, we generated a TF profile by choosing the top 20 candidates based on the number of genes they regulated. Then, we compared the TF profiles between different xylose utilization conditions in the host of either CTY or INVSc1, from which we selected the common TFs as the key modules in xylose metabolism regulation. A total of 15 TFs were found to be the key regulatory modules in the CTY host while 5 TFs were found in the INVSc1 host. Specifically, three TFs, Gcn4p, Rpn4p, and Yap1p, stood out as the regulatory modules that were always involved in the xylose metabolism regulation regardless of which xylose pathway was used and in which host the xylose pathway was expressed.

As indicated by GO analysis, the cellular amino acids process (GO:0006520) was found to be tightly regulated in both the CTY and INVSc1 hosts, which may explain the pivotal role Gcn4p played in xylose metabolism, since it is well known as a transcriptional activator of amino acids biosynthesis [12]. The Rpn4p and Yap1p have been reported as transcriptionally regulated under stressed conditions [13-16]. Rpn4p is one of the key transcriptional factors that control the degradation of damaged or aggregated proteins [17-19]. Considering the fact that the expression of heterologous proteins including XR, XDH, and XKS is required for xylose utilization in recombinant *S. cerevisiae*, one of the key functions of Rpn4p may be degrading the mis-folded heterologous proteins. In *S. cerevisiae*, the oxidative stress is primarily controlled by Yap1p. Since the fungal xylose pathway expressed in *S. cerevisiae* was cofactor imbalanced, NADH was produced when xylitol was converted to xylulose. The oxidative stress caused by NADH overproduction may trigger the transcriptional regulation of Yap1p [17-19]. According to this study so far, the xylose utilization in recombinant *S. cerevisiae* was indicated to involve both carbohydrate metabolism regulation and stress responses. However, in order to decode the more detailed regulatory patterns of the TFs involved in xylose metabolism, tremendous genotype-phenotype correlation experiments needs to be accomplished in future.

### Transcriptional characterization of host dependence in xylose utilization

**Figure 5:**
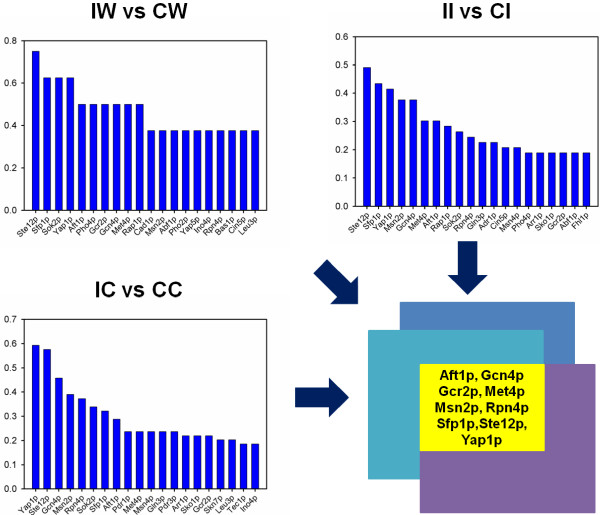
**TF profiles shown as the percentage of genes regulated by the top 20 TFs relative to the total number of genes involved in host dependence.** The highlighted TFs in the yellow box indicate the ones that appeared in all of the host dependence experiments.

The transcriptional behaviors of host dependence were characterized by comparing the global gene expression levels in the CTY and INVSc1 hosts in response to the same xylose pathway (Figure 3). When the WT pathway was utilized by the CTY and INVSc1 hosts respectively, eight genes were identified as down-regulated, while 59 genes (36 down-regulated and 23 up-regulated) and 63 genes (29 down-regulated and 34 up-regulated) were differentially expressed in different hosts when CTYp or INVp was used, respectively. The GO analysis indicated that the key biological processes involved in the host dependence include carbohydrate metabolic process (GO:0005975), nucleobase-containing small molecule metabolic process (GO:0055086), cofactor metabolic process (GO:0051186), generation of precursor metabolites and energy (GO:0006091), and cellular amino acid metabolic process (GO:0006520). Following the similar TF analysis as discussed previously, we found nine TFs as the key regulatory modules in host dependence (Figure 5). Among the nine TFs, three TFs (i.e., Gcn4p, Gcr2p, and Met4p) were transcriptional regulators of amino acids metabolisms, while four TFs (i.e., Msn2p, Rpn4p, Sfp1p, and Yap1p) were required by stress responses (Additional file 1: Table S1). The other two TFs, Aft1p and Ste12p, were involved in iron metabolism/homeostasis and signaling pathways, in carbon metabolism of *S. cerevisiae* respectively. Interestingly, the conserved TFs in xylose metabolism, Gcn4p, Rpn4p, and Yap1p, were also found among the key modules responsible for the host dependence, suggesting that the same regulatory modules may play different roles in the transcriptional regulation of carbon metabolism in different hosts of *S. cerevisiae*.

**Figure 6:**
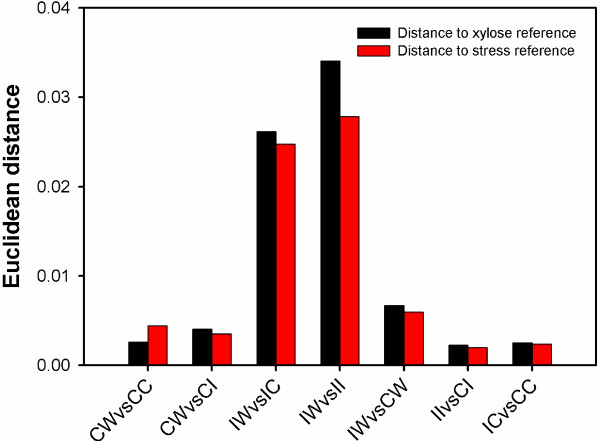
Euclidean distance of the sample TF profiles to two reference TF profiles: xylose reference profile (reflecting carbohydrate metabolism regulation) and stress reference profile (reflecting stress responses).

As indicated by the TF analysis, the transcriptional behaviors of xylose utilization in recombinant *S. cerevisiae* may be affected by both carbohydrate metabolism regulation and stress response. To deconvolute such two effects on xylose metabolism regulation, we solicited two transcriptional datasets from the GEO database as the reference datasets for xylose-related carbohydrate metabolism regulation (i.e., GSE27325) and stress responses (i.e., GSE3812), respectively. The top 250 genes which are differentially expressed in the reference datasets were extracted, followed by the TF analysis to generate the reference TF profiles. In order to make a direct comparison, both the reference TF profiles generated from GEO database and the sample TF profiles generated from this study were normalized, based on which the Euclidean distances were calculated (Figure 6). As a commonly used measure for the similarity between two profiles [20], the Euclidean distance could reflect the similarity between the sample TF profile and the reference TF profile. For the TF profiles of host dependence (i.e., IW vs. CW, II vs. CI, and IC vs. CC), the Euclidean distance to the reference TF profiles of xylose-related carbohydrate metabolism regulation was close to that of stress responses, supporting the hypothesis that both xylose regulation and stress responses may be involved in regulating xylose metabolism. In addition, compared to those in the CTY host (i.e., CW vs. CI and CW vs. CC), the Euclidean distances to the reference TF profiles of both carbohydrate metabolism regulation and stress response were nearly one magnitude larger in the INVSc1 host (i.e., IW vs. II and IW vs. IC), which indicated that diverse regulatory strategies could be adopted by different hosts. Interestingly, the optimization of the xylose pathway in the INVSc1 host led to a smaller Euclidean distance (~23%) to the stress response than the xylose utilization (i.e., IW vs. II), while on the other hand, the optimization of the xylose pathway in the CTY host led to a smaller Euclidean distance (~35%) to the carbohydrate metabolism regulation than the stress response (i.e., CW vs. CC). The discrepancy suggested that the direction of pathway optimization could be different between the CTY and INVSc1 hosts, since the better xylose fermentation behaviors in the INVSc1 host were more likely to be attributed to improved responses to environmental stresses, while regulating the pathways in the central carbon metabolism may be more crucial for the improved xylose utilization in the CTY host.

## Discussion

The xylose-based ethanol production in recombinant *S. cerevisiae* was affected by many factors, including the choice of heterologous pathway, the cultivation medium, and the oxygen availability. Previous studies [1,21] have found the optimal ethanol production can be achieved by cultivating recombinant *S. cerevisiae* strains in nutrient rich medium under oxygen limited conditions. Yet, the xylose pathways still need to be optimized to improve the titer and productivity of ethanol. Our laboratory has developed a combinational transcriptional engineering approach to screen and select the optimal pathways from thousands of mutated fungal xylose pathways with various combinations of XR, XDH, and XKS expressions [6]. By applying this pathway engineering approach in two *S. cerevisiae* hosts, two xylose pathways stood out as the optimal pathways in the corresponding host. However, the expression profiles of XR, XDH and XKS in these optimal pathways, CTYp and INVp, were not the same (Additional file 1: Figure S3). While the expression profiles of XR, XDH and XKS in INVp were similar as those in the wild-type pathway (WT), the XDH in CTYp always had a much lower expression level than that in WT. Such discrepancy could be resulted from the different strategies used by CTYp and INVp when optimizing xylose utilization. In the INV host, the xylose utilization was more likely to be improved by coordinating the heterologous pathway expression with the stress responses instead of the central metabolism, which led to minor adjustment of the XR, XDH and XKS expression profile. However, coordinating the heterologous pathway expression and the central metabolism could contribute largely to improve xylose utilization in the CTY host, which required the expression profile to be changed in the optimal pathway in order to be more suitable with the central carbon metabolism. The utilization of different metabolic strategies in the CTY and INV hosts was also consistent with the RNA-seq analysis, as few genes in the central metabolic pathways were differentially expressed when comparing the transcriptomic behaviors of INV-INVp and INV-WT while several key genes in the TCA cycle and lipid synthesis (e.g. CIT1 and ACC1) were found to be differentially expressed when comparing the transcriptomic behaviors of CTY-CTYp and CTY-WT. The host dependence thereby rises as a result of suboptimal combinations of the yeast hosts and the metabolic strategies. When switching the CTYp from the CTY host into the INV host, the stress responses of the INV host could not be enhanced and hence the xylose utilization was not improved. Similarly, when switching INVp from the INV host into the CTY host, the similar expression profile in the INVp as that in the WT did not benefit the coordination between the heterologous pathway expression and the central metabolism in the CTY host, which led to the low-performance in xylose utilization as that in CTY-WT. The host-dependence revealed by this study suggested that the harmony of pathway optimization and the inherent metabolic strategies used by the host strains would determine the success of metabolic engineering.

The genome-scale transcriptional analysis in this study has identified several genes that could play important roles in xylose utilization by different yeast hosts. Among all the genes in the CTY host involved in the coordination of the heterologous pathway expression with the central metabolism, CIT1 gene, encoding the citrate synthase in the TCA cycle, were identified in both the comparison of CTY-WT to CTY-CTYp and the comparison of CTY-WT to CTY-INVp. This could suggest that the TCA cycle was one of the key targets subject to transcriptional regulation for optimizing xylose utilization. In addition, ALD4 and ACC1 genes, encoding the aldehyde dehydrogenase and acetyl-CoA carboxylase, respectively, were only found to be differentially expressed when the optimal CTYp was used in the CTY host, which indicated that the synthesis and utilization acetyl-CoA could be related to improvement of the coordination between the heterologous pathway expression and the central metabolism. As for the INV host, the expression of suboptimal CTYp led to decrease of transcriptional level of SOD1, which is one of the key genes in response to the oxidative stress. Consequently, the stress response of INV-CTYp could be affected and become not as optimal as that INV-INVp, which led to poorer xylose utilization in INV host.

In this study, three transcription factors (Gcn4p, Yap1p, and Rpn4p) were selected as the conserved regulative modules for xylose metabolism. To further validate their indispensable role in regulating the xylose utilization regardless of which pathway was expressed and which host the xylose pathway was expressed in, we solicited two additional datasets from the published transcriptional studies. The first dataset included the differentially expressed genes in a recombinant *S. cerevisiae* growing with glucose or xylose as the carbon source [22]. The TF analysis (Additional file 1: Figure S4) showed that Gcn4p, Yap1p, and Rpn4p were among the top 20 TFs that were involved in regulating yeast metabolism in response to xylose utilization. The second dataset investigated the transcriptional behaviors of recombinant *S. cerevisiae* strains harboring a xylose isomerase pathway under xylose utilization conditions. Compared to the fungal xylose pathway used in this study, the xylose isomerase pathway does not have the cofactor imbalance issue [2,23]. However, the three TFs identified in transcriptional analysis for the fungal xylose pathway were also discovered as the key regulatory module in xylose utilization via xylose isomerase pathway (Additional file 1: Figure S5). Combining all the evidences together, the conserved role of Gcn4p, Yap1p, and Rpn4p in regulating xylose metabolism was validated.

## Conclusions

The xylose utilization in recombinant *S. cerevisiae* depends not only on the choice of the heterologous pathway but also the choice of the host. To perform a systematic investigation of the so-called host dependence, we applied RNA-seq analysis in this study to characterize the transcriptional behaviors of six strains, created by expressing three xylose pathways in two hosts. We identified three transcription factors as the conservative modules that regulated the xylose metabolism regardless of which xylose pathway was used and which host the xylose pathway was expressed in. Another nine transcription factors were found as the key regulatory modules playing pivotal roles in the host dependence. Based on the transcription factor analysis, xylose utilization in recombinant *S. cerevisiae* may involve both carbohydrate metabolism regulation and stress responses. The diverse regulatory strategies and the different directions of pathway optimization in the context of various *S. cerevisiae* hosts are hypothesized to cause the host-specific responses to xylose utilizations. In sum, the work presented in this study can be viewed as a stepping stone towards a more comprehensive understanding of the regulatory machinery of the cellulosic sugar metabolism in recombinant *S. cerevisiae*, and provide valuable insights towards improved engineering strategies for cellulosic ethanol production.

## Material and methods

### Strains, media, and culture conditions

The parent *S. cerevisiae* strain INVSc1 (*MATa his3Δ1 leu2 trp1-289 ura3-52 MATα his3Δ1 leu2 trp1-289 ura3-52*) was purchased from Invitrogen (Life Technologies, Grand Island, NY, USA). Still Spirits (Classic) Turbo Distiller’s Yeast (CTY) was purchased from Homebrew Heaven (Everett, WA, USA). Three xylose pathways, namely WT (the wild-type pathway without optimization of promoter strengths), CTYp (the xylose pathway with optimization of promoter strengths in the CTY host), and INVp (the xylose pathway with optimization of promoter strengths in the INV host), were constructed previously using the COMPACTER approach [6]. The plasmids with the three xylose pathways were then transformed into the CTY and INVSc1 hosts to create six recombinant strains, named as CTY-WT (i.e., CTY host with the WT pathway), CTY-CTYp (i.e., CTY host with the CTYp pathway), CTY-INVp (i.e., CTY host with the INVp pathway), INV-WT (i.e., INV host with the WT pathway), INV-CTYp (i.e., INV host with the CTYp pathway), and INV-INVp (i.e., INV host with the INVp pathway). All yeast strains were stored in 25% glycerol at −80°C. To culture *S. cerevisiae* strains, seed cultures were grown in YPAD media (1% yeast extract, 2% peptone, 0.01% adenine hemisulfate, 2% glucose) at 30°C overnight. The seed cultures were then inoculated (1%, v/v) into the YPAX medium with 4% xylose as the carbon source. All of the yeast strains were cultivated at 30°C and 100 rpm for oxygen limited conditions, with initial cell concentration at ~0.08 g DCW/L. Three biological replicates were made when culturing each of the six recombinant strains.

### RNA preparation

Samples were taken at the log phase of the six recombinant strains (~24 h). The cell pellets (~10 mg) were frozen by liquid nitrogen. Total RNA was extracted by FastRNA Spin kit for yeast (MP Biomedicals) according to the manufacturer’s instructions. The RNA quality and quantity were determined using Agilent 2100 Bioanalyzer (Agilent Technologies, Santa Clara, CA, USA). The RNA integrity number (RIN) of all RNA samples used for sequencing was more than 9.0. The RNA samples were then sent to The Biotechnology Center at University of Illinois at Urbana-Champaign for library preparation and sequencing.

### RNA-seq library preparation and sequencing

RNA-seq libraries were constructed and sequenced at 7the W. M. Keck Center at the University of Illinois at Urbana-Champaign. Eighteen libraries were constructed using the TruSeq RNA Sample Preparation Kit (Illumina, San Diego, CA, USA). Briefly, mRNA was selected from total RNA with oligo dT beads and chemically fragmented. First-strand cDNA was synthesized with random hexamer primers and SuperScript II (Life Technologies). Double stranded DNAs were blunt-ended, 3′-end A-tailed and ligated to indexed adaptors. The adaptor-ligated double-stranded cDNA were amplified by PCR for 10 cycles with the Kapa HiFi polymerase (Kapa Biosystems, Woburn, MA) to reduce the likelihood of multiple identical reads due to preferential amplification. The final libraries were quantitated with Qubit (Life Technologies, Grand Island, NY) and the average size was determined on an Agilent bioanalyzer DNA7500 DNA chip (Agilent Technologies, Santa Clara, CA, USA) and diluted to 10 nM. The 10 nM dilution was quantitated by qPCR on an ABI 7900 Real-time PCR system (Life Technologies).

The libraries were pooled in equimolar concentration and loaded onto 8-lane flowcells for cluster formation and sequenced on an Illumina HiSeq2000. The libraries were sequenced from both ends of the DNA molecules to a total read length of 100 nt from each end. The output from the lane with 18 libraries was 442,365,348 reads.

### Transcriptional analysis of RNA-seq data

The sequence files in FASTQ format were analyzed using the Galaxy software (http://usegalaxy.org). Briefly, the files were groomed to make sure the quality-scores line in the files use Sanger-scaled quality values with ASCII offset 33. The RNA-seq paired-end reads were mapped into transcripts using TopHat by setting the reference genome as *S. cerevisiae* (sacCer3, UCSC). The transcripts were assembled and the FPKM (fragments per kilobase of exon per million fragments mapped) were estimated using Cufflinks with the default parameter settings, followed by transcripts merge using Cuffmerge. The assembled transcripts between control group and experimental group were compared using Cuffdiff, with cutoff p-value set as 0.05. The transcripts, of which the FPKM were identified as significantly different between the control group and experimental group, were picked and searched in the genome browser of BioCyc database to identify the specific genes included in the transcripts. The transcripts cluster analysis was achieved by using ‘clustergram’ of MATLAB (MathWorks, Natick, MA, USA). The gene ontology analysis was performed by using generic GO term mapper developed by Princeton University (http://go.princeton.edu/cgi-bin/GOTermMapper).

### Transcriptional factor analysis

To generate the regulation matrix, the YEASTRACT database (http://www.yeastract.com/) [24] was solicited, in which the differentially expressed genes identified by the RNA-seq analysis were searched against all of the transcription factors (TFs) in the YEASTRACT database (only documented regulations with direct or indirect evidences were taken into consideration). To provide the TF profiles, the number of genes that a TF can regulate in the pool of genes that were found to be differentially expressed was calculated by YEASTRACT database. Then, it is divided by the total number of genes that were found to be differentially expressed. To provide the normalized TF profiles, the TF profiles was generated with an additional normalization as $TF_{\mathrm{norm}}=\frac{\mathrm{TF}}{\left|\mathrm{TF}\right|}$, where *TF* and *TF*_*norm*_ are the un-normalized and normalized transcription factor profiles, respectively. To get the reference and normalized transcriptional factor profiles for xylose metabolism and stress responses, two additional transcriptional datasets (ID: GSE27325 and GSE3812) were obtained from Gene Expression Omnibus (GEO) (http://www.ncbi.nlm.nih.gov/geo/). The top 250 differentially expressed genes were extracted and passed through the similar flowchart as described above to generate the reference normalized TF profile for xylose metabolism and stress responses, respectively. The Euclidean distance of the sample normalized TF profiles to the reference normalized TF profiles was then calculated as $d=\sqrt{\sum_{i=1}^{n}{\left(TF_{i,s}-TF_{i,r}\right)}^{2}}$, where *TF*_*i,s*_ and *TF*_*i,r*_ are the *i*th element in the sample normalized TF profile and reference normalized TF profile, respectively, and *n* is the number of elements in the normalized TF profile.

## Abbreviations

XR: Xylose Reductase (XR); XDH: Xylitol Dehydrogenase (XDH); XKS: Xylulose Kinase; COMPACTER: Customized Optimization of Metabolic Pathways By Combinatorial Transcriptional Engineering; TFs: Transcription Factors; FPKM: Fragments Per Kilobase of Exon Per Million Fragments Mapped; GO: Gene Ontology; qPCR: quantitative Polymerase Chain Reaction; GEO: Gene Rxpression Omnibus; YPAD: Yeast Extract Peptone Dextrose; YPAX: Yeast Extract Peptone Xylose; DCW: Dry Cell Weight; CW: CTY-WT (i.e., CTY host with the WT pathway); CI: CTY-INVp (i.e., CTY host with the INVp pathway); CC: CTY-CTYp (i.e., CTY host with the CTYp pathway); IW: INV-WT (i.e., INV host with the WT pathway); II: INV-INVp (i.e., INV host with the INVp pathway); IC: INV-CTYp (i.e., INV host with the CTYp pathway).

## Competing interests

The authors declare that they have no competing interests.

## Authors’ contributions

XF carried out all the experiments, analyzed the data and drafted the manuscript. XF and HZ read, revised and approved the final manuscript.

## Supplementary Material

Additional file 1: Table S1 Description of transcriptional factors involved in context dependence. **Figure S1.** Correlation between the cut-off valueand the number of transcripts identified as differentially expressed. **Figure S2.** Cluster analysis of transcriptional responses of INVSc1 host to xylose metabolism. **Figure S3.** FPKM of XR, XDH, and XKS in different recombinant *S. cerevisiae* strains. **Figure S4.** TF profiles of xylose metabolism regulations as reported in Microbial Cell Factories 2008, 7:18. **Figure S5.** TF profiles of xylose metabolism regulations using xylose isomerase pathway. Click here for file
