# Supplementary material for: Investigating host dependence of xylose utilization in recombinant Saccharomyces cerevisiae strains using RNA-seq analysis
Source: Biotechnol Biofuels. 2013 Jul 6;6:96. doi: 10.1186/1754-6834-6-96 (PMC3706274; doi:10.1186/1754-6834-6-96)
Supplement: Additional file 1: Table S1 — Description of transcriptional factors involved in context dependence. Figure S1. Correlation between the cut-off valueand the number of transcripts identified as differentially expressed. Figure S2. Cluster analysis of transcriptional responses of INVSc1 host to xylose metabolism. Figure S3. FPKM of XR, XDH, and XKS in different recombinant S. cerevisiae strains. Figure S4. TF profiles of xylose metabolism regulations as reported in Microbial Cell Factories 2008, 7:18. Figure S5. TF profiles of xylose metabolism regulations using xylose isomerase pathway. [file 1754-6834-6-96-S1.docx]

**Supplementary Data**

**Investigating host dependence of xylose utilization in recombinant *Saccharomyces cerevisiae* strains using RNA-seq analysis**

Xueyang Feng, Huimin Zhao

**Figure S1** The number of transcripts identified with different cut-off values (n = 5-9) of the comparisons in which the target fragments maintain consistent transcriptional behaviors.





**Figure S2** FPKM of XR (black bars), XDH (red bars), and XKS (green bars) in different recombinant *S. cerevisiae* strains.

B

A

**Figure S3** (A) FPKM between INV-WT (IW) and INV-CTYp (IC), in which R1, R2, and R3 indicate three biological replicates; (B) FPKM between INV-WT (IW) and INV-INVp (II), in which R1, R2, and R3 indicate three biological replicates.





**Figure S4** TF profiles shown as the percentage of genes regulated by the top 20 TFs relative to the total number of genes involved in xylose metabolism regulations as reported in *Microbial Cell Factories* 2008, 7:18.


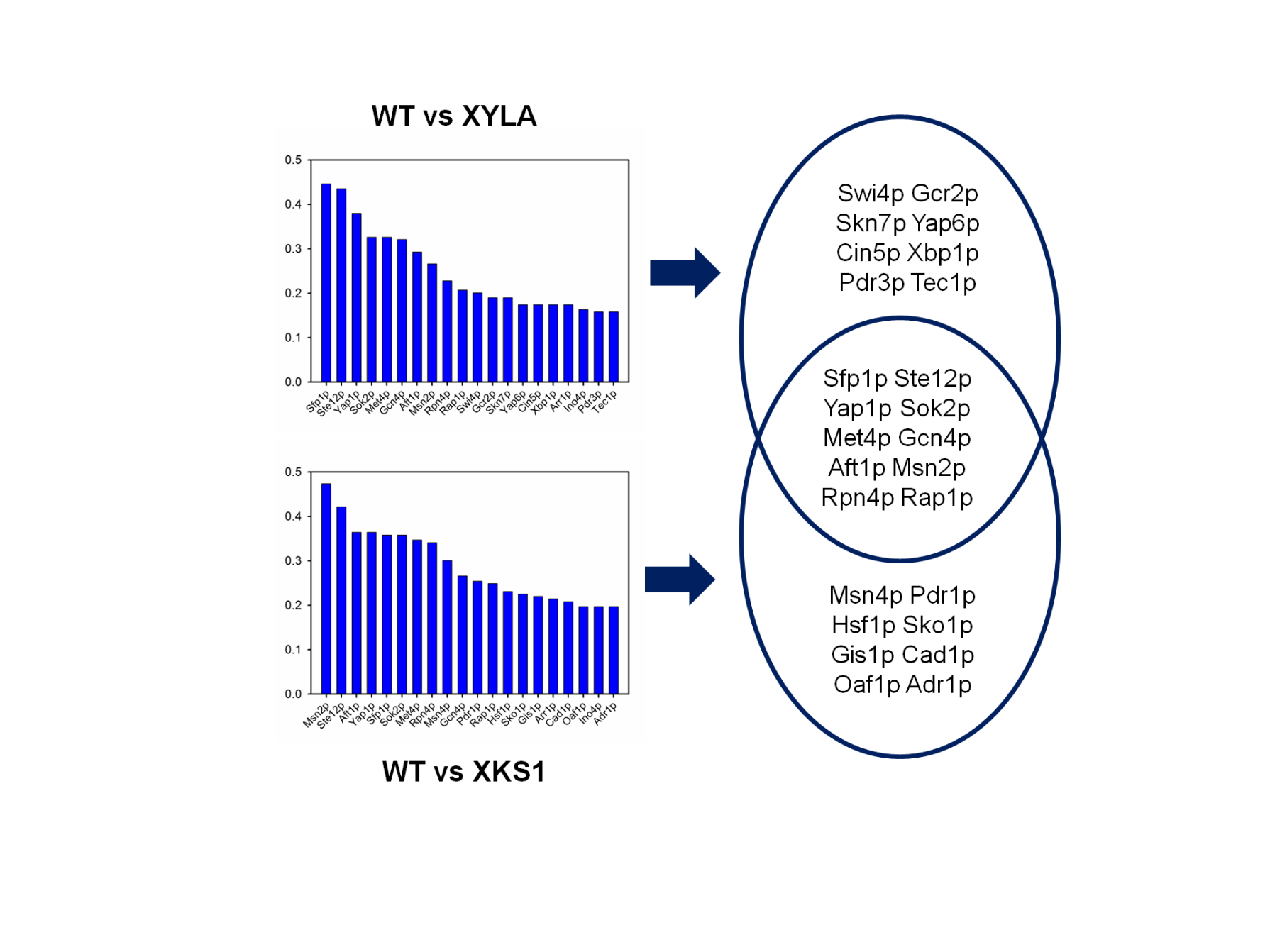


**Figure S5** TF profiles shown as the percentage of genes regulated by the top 20 TFs relative to the total number of genes (i.e., the top 250 differentially expressed genes extracted from GSE27325) involved in xylose metabolism regulations using the xylose isomerase pathway. WT vs XYLA: the wild-type strain treated with xylose compared to the wild-type strain with introduction of the heterologous XYLA gene; WT vs. XYLA: the wild-type strain treated with xylose compared to the wild-type strain with introduction of the heterologous XYLA gene and XKS1 gene.

**Table S1** Description of transcriptional factors involved in context dependence

| **Transcriptional factor** | **Description (from YEASTRACT database)** |
| --- | --- |
| Aft1p | Transcription factor involved in iron utilization and homeostasis; binds the consensus site PyPuCACCCPu and activates the expression of target genes in response to changes in iron availability. |
| Gcn4p | Transcriptional activator of amino acid biosynthetic genes in response to amino acid starvation; expression is tightly regulated at both the transcriptional and translational levels. |
| Gcr2p | Gcr1p and Gcr2p are transcriptional activators of glycolytic genes in *Saccharomyces cerevisiae*. Gcr1p is a DNA-binding protein interacting with the consensus sequence CTTCC, whereas Gcr2p interacts with Gcr1p. |
| Met4p | Leucine-zipper transcriptional activator, responsible for the regulation of the sulfur amino acid pathway, requires different combinations of the auxiliary factors Cbf1p, Met28p, Met31p and Met32p. |
| Msn2p | Transcriptional activator related to Msn4p; activated in stress conditions, which results in translocation from the cytoplasm to the nucleus; binds DNA at stress response elements of responsive genes, inducing gene expression. |
| Rpn4p | Transcription factor that stimulates expression of proteasome genes; Rpn4p levels are in turn regulated by the 26S proteasome in a negative feedback control mechanism; RPN4 is transcriptionally regulated by various stress responses. |
| Sfp1p | Regulates transcription of ribosomal protein and biogenesis genes; regulates response to nutrients and stress, G2/M transitions during mitotic cell cycle and DNA-damage response, and modulates cell size; regulated by TORC1 and Mrs6p; sequence of zinc finger, ChIP localization data, and protein-binding microarray (PBM) data, and computational analyses suggest it binds DNA directly at highly active RP genes and indirectly through Rap1p at others. |
| Ste12p | Transcription factor that is activated by a MAP kinase signaling cascade, activates genes involved in mating or pseudohyphal/invasive growth pathways; cooperates with Tec1p transcription factor to regulate genes specific for invasive growth. |
| Yap1p | Basic leucine zipper (bZIP) transcription factor required for oxidative stress tolerance; activated by H_2_O_2_ through the multistep formation of disulfide bonds and transit from the cytoplasm to the nucleus; mediates resistance to cadmium. |
